# Effectiveness of psychological interventions for treating chronic prostatitis/chronic pelvic pain syndrome: A protocol for systematic review and meta-analysis

**DOI:** 10.1097/MD.0000000000022151

**Published:** 2020-09-25

**Authors:** Yuanjie Xu, Ling Zhang, Yifeng Shen, Hangyu Yao, Shanshan Yong, Yaodong You

**Affiliations:** a Chengdu University of Traditional Chinese Medicine; b Hospital of Chengdu University of Traditional Chinese Medicine, Chengdu, Sichuan Province, China.

**Keywords:** chronic prostatitis/chronic pelvic pain syndrome, protocol, psychological

## Abstract

**Introduction:**

Chronic prostatitis/chronic pelvic pain syndrome (CP/CPPS) is one of the most common diseases in urology, which 50% of men are infected at some point in their lives. Type III CP/CPPS is the most complex and controversial of all types of prostatitis, the highest incidence rate, uncertain efficacy, the long-term treatment that affects the patient's psychopathic symptoms, increases the psychological burden of patients. Psychological intervention for patients with CP/CPPS, which is difficult to treat with drugs and physics, can effectively improve clinical efficacy and improve the psychological condition. The researchers found a high prevalence of psychosocial problems and catastrophic distress in CP/CPPS patients, such as serious mental disorders, especially depression, anxiety and stress, and the high incidence of pain-devastating illness. In this study, we will evaluate psychological interventions as an effective way to relieve chronic prostatitis.

**Methods and analysis:**

The databases of English databases (PubMed, MEDLINE, EMBASE, Web of Science, Cochrane Library) and Chinese databases (China National Knowledge Infrastructure, China Biology Medicine Database, Wanfang Database, VIP Database) will be retrieved. The search strategy that will be run in the PubMed and tailored to the other database when necessary is presented in this article. RevMan 5.3 and Stata 11.0 will be used for Systematic Review and Meta-analysis. This protocol reported under the Preferred Reporting Items for Systematic Reviews and Meta-Analyses Protocols (PRISMA-P) statement, and we will report the systematic review by following the PRISMA statement.

**Results:**

The study is a protocol for systematic review and meta-analysis without results, and data analysis will be carried out after the protocol. We will share our findings in the third quarter of 2021.

**Conclusion:**

This systematic review will provide more evidence to assess whether psychological is an effective intervention for patients with chronic prostatitis/chronic pelvic pain syndrome. Besides, the results will be published in a public issue journal and offer the urologists help to make clinical decisions.

**Ethics and dissemination:**

Formal ethical approval is not required in this protocol. We will collect and analyze data based on published research. Since this research does not involve patients, personal privacy will not be affected. The results of this review will be distributed to peer-reviewed journals or submitted to relevant conferences.

**Protocol registration number:**

INPLASY202080021

## 1 Introduction

Prostatitis is an important global problem. The incidence of prostatitis-like symptoms ranges from 2% to 9.7%, with an average incidence of 8.2%.^[1]^ CP/CPPS accounts for 90% to 95% of prostatitis cases,^[2]^ CP/CPPS is usually associated with low sexual, erectile dysfunction,^[3]^ painful ejaculation, psychological disorders and decreased semen quality,^[4]^ and so on. More than 80% of patients have some psychological problems, and 20%-50% of them have serious psychological problems.^[5]^ Seriously it may affect the quality of life (QoL) and even lead to divorce.^[6]^

The National Institute of Health (NIH) divides prostatitis into Type I–IV, and Type III CP/CPPS is divided into inflammatory chronic pelvic pain syndrome (IIIa) and noninflammatory chronic pelvic pain syndrome (IIIb)^[7]^ and the National Institute of Health - Chronic Prostatitis Symptom Index (NIH-CPSI) score is a validated measure commonly used to measure CP/CPPS symptoms.^[8]^ After that, the UPOINT clinical phenotyping system, developed by Shoskes et al, can be used to carry out special clinical classification of CPPS.^[9]^ CP/CPPS is a chronic disease with a variety of psychosocial and physical symptoms. It is a syndrome caused by different reasons, such as nonbacterial microbial infection, urine reflux, neuroendocrine factors, autoimmune, psychological factors, oxidative stress (OS),^[10,11]^ and chronic pelvic congestion and other factors. CP/CPPS patients show Th1 and Th17 immune responses against Prostate antigen (Pag). These immune responses may be the basis for the induction and development of chronic pelvic pain and inflammation of the male reproductive tract. These and produce excessive reactive oxygen species by oxidative stress can damage prostate function, affect semen quality, sperm concentration, decreased mobility and vitality, and increase sperm apoptosis.^[4,12,13]^ Besides, the long-term treatment affects the patient's mental and psychological symptoms and increases the patient's psychological burden. Changes in these mental and psychological factors can cause nonautonomous nerve dysfunction, resulting in neuromuscular dysfunction in the posterior urethra, leading to pelvic pain and urinary dysfunction, thereby aggravated chronic prostatitis.

At present, more and more attention has been paid to the psychological intervention treatment of type III prostatitis. People have different feelings about pain. Chronic pain usually leads to depression or catastrophic thinking. Too much focus on symptoms may increase pain.^[14,15]^ Psychological intervention treatment of CP/CPPS patients who are difficult to treat with drugs and physics can effectively improve the clinical efficacy and improve the psychological status.^[16]^

## 2 Objectives

With this systematic review and if possible, meta-analysis we urge to further evaluate the effectiveness of psychological as a way to alleviate chronic prostatitis/chronic pelvic pain. The results will offer clinical decisions for urologists and andrologists. So far, the meta-analysis about the effect of CP/CPPS suggested that psychological may be a possible treatment for CP/CPPS; however, more studies with appropriate controls are needed to confirm this finding. Further investigation is warranted given that an increasing number of studies about the effects of psychological intervention for CP/CPPS have been carried out in recent years. Therefore, we will conduct an up-to-date systematic review and meta-analysis for existing RCTs to further assess the effectiveness of the psychological intervention as a way to alleviate CP/CPPS and improve QOL.

## 3 Methods

The protocol was registered on the International Platform of Registered Systematic Review and Meta-analysis Protocols (registration number: INPLASY202080021) which could be available on https://inplasy.com. The content refers to the statement of preferred reporting items for systematic review and meta-analysis protocols (PRISMA-P).

### 3.1 Eligibility criteria

The inclusion and exclusion criteria are as follows.

#### 3.1.1 Types of studies

All the RCTs of CP/CPPS patients who were treated by psychological interventions will be included without publication status restriction or writing language. In cases of duplication (i.e., publications of the same sample), we will include the publication with the largest sample size. If sample sizes reported in 2 manuscripts are the same, the first published study will be included in the review. Qualitative studies that explore the acceptability of the intervention will also be included. All identified articles will be screened despite their publication dates and set of interventions.

#### 3.1.2 Participants

Inclusion criteria:

- pain in penis, testicles, perineum, or lumbosacral region.
- voiding symptoms, such as dysuria, frequency, and sense of incomplete urination.
- prostatic fluid, semen, and urine bacterial culture were negative.
- the minimum duration of these symptoms for inclusion in the study was 3 months.

Exclusion criteria:

- patients with a past medical history for documented urinary tract infections and sexually transmitted diseases.
- patients with urethral malformation and stricture, urinary calculi, and neurogenic bladder.
- patients with benign prostatic hyperplasia, epididymitis, and spermatic cord disease.
- patients with neurological disease (vertebral column disease, trauma or surgery, disease affecting nervous system, etc.)
- patients with mental illness

#### 3.1.3 Types of interventions and controls

Experimental interventions:

The intervention received may be a psychotherapeutic intervention that was facilitated through a specialized program, or by a registered psychologist, licensed therapist, or other trained and licensed professional credentialed to provide specific counseling. The patients in the treatment group received psychological intervention therapy (include psychological counseling, psychotherapy, psychological support, and psychoeducation, no limit on-one psychotherapy and group therapy and facilitated in person, on the telephone, online, or via distance delivery (method of delivery).

Control interventions:

The control group could gain routine drug medications or guideline-recommended conventional treatment and health education.

#### 3.1.4 Types of outcome measures

Primary outcome:

1) NIH-CPSI scores decreased (NIH-CPSI to evaluate the patient's symptom score before and after treatment, the scale has a total score of 43 points, including pain or discomfort (21 points), urinary symptoms (10 points), and quality of life (12 points), the higher the score, the more severe the symptoms).

Secondary outcomes:

1. scores of IIEF-5
2. SAS scores, SDS scores (The Anxiety Scale Rating Scale (SAS), and Self-Rating Depression Scale (SDS) are used to assess the patient's psychological status. The total score of both scales is 100 points. The higher the score, the worse the patient's psychological status.)
3. Quality of Life Comprehensive Assessment Questionnaire (QOL)

### 3.2 Search strategy

#### 3.2.1 Data sources

Electronic databases will include English databases (PubMed, MEDLINE, EMBASE, Web of Science, Cochrane Library) and Chinese databases (China National Knowledge Infrastructure, China Biology Medicine Database, Wan fang Database, VIP Database). All the above databases will be searched from their inception to recognize related studies. The search strategy that will be run in the PubMed and tailored to the other database when necessary is presented in Table 1. Besides, the reference lists of review articles will be searched for any possible titles matching the inclusion criteria.

**Table 1:**
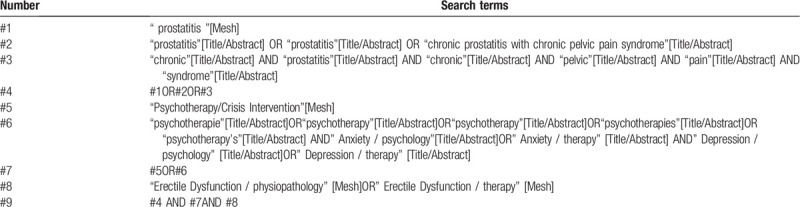
This table presents the initial draft of the search strategy with PubMed as an example.

#### 3.2.2 Other sources of search

The researchers will also scan the database of Chengdu University of Traditional Chinese Medicine Library and consult the experts in urology. Dissertations of degrees will be included. The WHO International Clinical Trials Registry Platform and Google Scholar will be scrutinized for potential results. Besides, the ClinicalTrials.govregistry will be explored to find any unpublished trials.

### 3.3 Data extraction, quality, and validation

#### 3.3.1 Study inclusion

**Figure 1:**
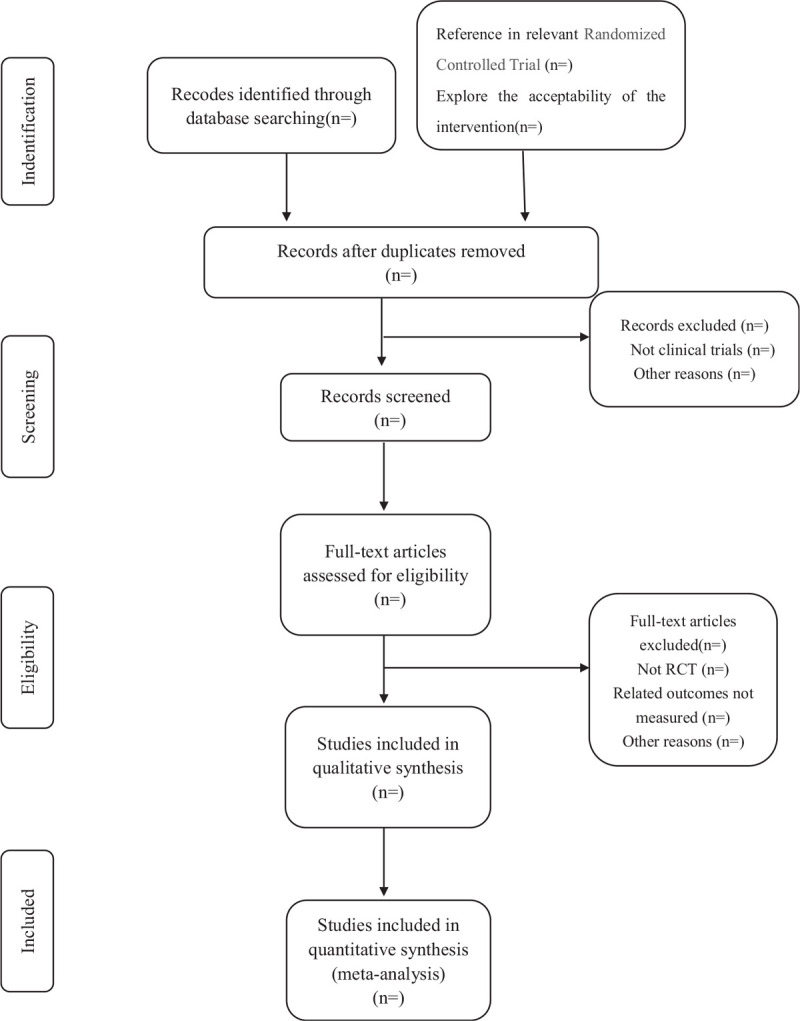
Study selection flow chart. The Preferred Reporting Items for Systematic Review and Meta-analysis Protocols (PRISMA) literature screening flow chart.

According to predefined eligibility criteria, researchers will import the literature retrieved to the Endnote X8 and eliminate the duplicate data. Studies will be removed if they do not meet the inclusion criteria. If the studies appear to meet the inclusion criteria or there is any uncertainty based on the information provided in the title and abstract, full texts will be obtained for further assessment. When necessary, we will contact the author for more details of the study to solve questions about eligibility. Two researchers will independently conduct the literature search and literature screening. Disagreements will be resolved by discussion or taking the expert (YY) for arbitration. The number and reasons for excluding trials will be recorded in detail. A flow diagram of the study selection is shown in Figure 1.

#### 3.3.2 Data extraction and management

Upon completion of the retrieval, the 2 reviewers will independently read and extract the data from the study. Data will include the following information: title, abstract, first author and corresponding author, the country, the publication time, publications, participants, demographic characteristics (age, marital status, course of illness, and education), the number of participants, diagnostic criteria, types, intervention format (online, individual, or group), study design (randomized vs nonrandomized), length of intervention and methodological quality of the study, observation index (NIH-CPSI, IIEF-5, SAS, SDS), the results of the study, the incidence of adverse events and type. We will use a standardized data extraction table to extract the above data. Any disagreement between the 2 reviewers will be decided by consensus or with the participation of a third reviewer. Finally, we will contact the author via email to request any missing data or clarification. If we cannot obtain the missing data, we will report it in the risk assessment of bias and consider its impact on the analysis of the data.

### 3.4 Risk of bias assessment

The risk of bias will be independently assessed by 2 reviewers and any differences will be resolved through consultation or the participation of a third reviewer. The RCTs will be evaluated using the Cochrane “risk of bias assessment” tool. The tool assesses the risk of bias mainly in the following 7 aspects: random sequence generation, allocation concealment, the blinding method for patients, researchers and outcomes assessors, incomplete result data, and selective reports. As recommended by the Cochrane manual, the risk of bias in each of these areas will be assessed as low or high depending on whether the criteria were met or not met, and the lack of information will be recorded as unclear. In most cases, disagreements will be settled by discussion between the 2 reviewers. If disagreement remained after discussion, a third reviewer will be consulted before taking the final decision on the disagreements.

### 3.5 Quantitative data synthesis and statistical methods

#### 3.5.1 Data analysis and synthesis

We will use RevMan5.3 software for meta-analysis. For dichotomous data (e.g., effective and ineffective), we will calculate risk ratio and 95% confidence intervals (CIs). For continuous data, when the measurement method and unit are consistent, we will calculate the weighted mean difference and 95% CIs. When the measurement methods and units are inconsistent or the mean values of different experiments differ greatly, we will use the standardized mean difference and 95% CIs as the composite statistics.

#### 3.5.2 Investigation of heterogeneity

Heterogeneity was evaluated with χ^2^ test results and I^2^ statistics.^[17]^ If *P*≤.10 or I2≥50%, heterogeneity will be considered significant. At this point, we will use the random-effects model and conduct meta-regression or sensitivity analysis to judge the robustness of the combined results and find out the source of heterogeneity.

#### 3.5.3 Subgroup analysis

If there is significant heterogeneity in the included trials, we will identify the source of heterogeneity through subgroup analysis and manage the heterogeneity:

1. The duration and severity of CP/CPPS.
2. The severity and duration of the patient's psychological condition.
3. Demographic characteristics of the patients: age, marital status, course of illness, and education.

#### 3.5.4 Sensitivity analysis

A sensitivity analysis will be performed to test the robustness of the review result and to detect the source of heterogeneity. This can be done by excluding trials with a high risk of bias or eliminating each study individually. And, the impact of methodological quality, sample size, and missing data will be assessed. Then the analysis will be repeated after the exclusion of low methodological quality studies and the results compared with the previous meta-analysis.

#### 3.5.5 Grading the quality of evidence

Grading of Recommendations Assessment, Development and Evaluation (GRADE) method^[18]^ will be performed to evaluate the level of confidence in regards to outcomes. It is based on 5 key domains: risk of bias, consistency, directness, precision, and publication bias. Two independent reviewers will assess these studies. In most cases, disagreements were resolved by discussion between the 2 reviewers. If disagreement remained after discussion, the third reviewer will be consulted before taking the final decision on the disagreements.

#### 3.5.6 Publication bias

Published bias will be measured by the funnel plot. If the result is indistinct, the Begg test and Egger test will be used (by STATA software 11.0).

#### 3.5.7 Reporting of the review

The methodological quality of the systematic review and meta-analysis will be standardized by each item of the AMSTAR-2 tool.^[19]^ And the results will be reported following the Preferred Reporting Items for Systematic Reviews and Meta-Analysis (PRISMA) statement.^[20]^

## 4 Discussion

Although there are many published articles and randomized controlled studies on various treatments, the etiology of CP/CPPS is still not clearly defined.^[21]^ The disease has a recurrent, the course stretches and other characteristics, the most common symptom is pain and lower urinary tract symptoms. Because the symptoms are concentrated in the lower abdomen, coupled with the lack of knowledge and misunderstanding of the disease, there are concerns about sexual life and fertility problems, and most patients are accompanied by psychological problems such as anxiety, depression, and nervousness. UPOINT-guided multimodal strategy treatment can provide more targeted treatment and provide better results than single-agent treatment. It has been verified in Europe and China.^[22–24]^ After the treatment of CP/CPPS patients, we found that emotional stress and depression are a potentially important factor in the development and prolongation of CP/CPPS. The elimination of mental stress can relieve symptoms or heal.

Therefore, clinical treatment should not only improve the symptoms of patients but also solve the mental and psychological problems of patients accordingly. Through this study, more detailed observation and analysis of patients with psychological interventions for CP/CPPS can guide the urologists to choose the treatment method more reasonably and concretely and adopt the most suitable treatment. There are some restrictions on this comment. As we are not good at other languages, the literature we search for is limited to Chinese and English, which will cause some prejudice. Besides, the limitation of the sample size also leads to the instability of the reliability of the conclusion.

## Author contributions

**Conceptualization:** Yuanjie Xu

**Data curation:** Yuanjie Xu, Ling Zhang

**Formal analysis:** Ling Zhang, Yifeng Shen

**Methodology:** Yuanjie Xu

**Project administration:** Yuanjie Xu, Ling Zhang, Yaodong You

**Software:** Hangyu Yao, Shanshan Yong

**Supervision:** Yaodong You

**Validation:** Yuanjie Xu, Ling Zhang

**Writing – original draft:** Yuanjie Xu

**Writing – review & editing:** Yaodong You
